# Employing a sequential multiple assignment randomized trial (SMART) to evaluate the impact of brief risk and protective factor prevention interventions for American Indian Youth Suicide

**DOI:** 10.1186/s12889-019-7996-2

**Published:** 2019-12-12

**Authors:** Victoria M. O’Keefe, Emily E. Haroz, Novalene Goklish, Jerreed Ivanich, The Celebrating Life Team, Mary F. Cwik, Allison Barlow

**Affiliations:** Johns Hopkins Bloomberg School of Public Health, Department of International Health, Center for American Indian Health, 415 N. Washington Street, 4th Floor, Baltimore, MD 21231 USA

**Keywords:** American Indian, Native American, Suicide, Resilience, Risk-reduction, Study design

## Abstract

**Background:**

This study is built on a long-standing research partnership between the Johns Hopkins Center for American Indian Health and the White Mountain Apache Tribe to identify effective interventions to prevent suicide and promote resilience among American Indian (AI) youth. The work is founded on a tribally-mandated, community-based suicide surveillance system with case management by local community mental health specialists (CMHSs) who strive to connect at-risk youth to treatment and brief, adjunctive interventions piloted in past research.

**Methods:**

Our primary aim is to evaluate which brief interventions, alone or in combination, have the greater effect on suicide ideation (primary outcome) and resilience (secondary outcome) among AI youth ages 10–24 ascertained for suicide-related behaviors by the tribal surveillance system. We are using a Sequential Multiple Assignment Randomized Trial with stratified assignment based on age and suicidal-behavior type, and randomizing *N =* 304 youth. Brief interventions are delivered by AI CMHSs, or by Elders with CMHS support, and include: 1) New Hope, an evidence-based intervention to reduce immediate suicide risk through safety planning, emotion regulation skills, and facilitated care connections; and 2) Elders’ Resilience, a culturally-grounded intervention to promote resilience through connectedness, self-esteem and cultural identity/values. The control condition is Optimized Case Management, which all study participants receive. We hypothesize that youth who receive: a) New Hope vs. Optimized Case Management will have significant reductions in suicide ideation; b) Elders’ Resilience vs. Optimized Case Management will have significant gains in resilience; c) New Hope followed by Elders’ Resilience will have the largest improvements on suicide ideation and resilience; and d) Optimized Case Management will have the weakest effects of all groups. Our secondary aim will examine mediators and moderators of treatment effectiveness and sequencing.

**Discussion:**

Due to heterogeneity of suicide risk/protective factors among AI youth, not all youth require the same types of interventions. Generating evidence for what works, when it works, and for whom is paramount to AI youth suicide prevention efforts, where rates are currently high and resources are limited. Employing Native paraprofessionals is a means of task-shifting psychoeducation, culturally competent patient support and continuity of care.

**Trial registration:**

Clinical Trials NCT03543865, June 1, 2018.

## Background

American Indian/Alaska Native (AI/AN) suicide inequities are concentrated in youth, with the highest rates among 10 to 24 year olds [1]. Recent data indicate that AI/AN youth and young adults (10–24) have suicide mortality rates (crude rate 27.09 per 100,000) that are more than 2 to 3 times higher same-aged European American (crude rate 12.60 per 100,000), African American (crude rate 7.97 per 100,000), and Asian/Pacific Islander (crude rate 8.68 per 100,000) peers [2]. These rates are likely underestimates due to jurisdictional challenges for who is responsible for reporting deaths when tribal lands intersect with county and state, racial and ethnic misclassification [1], and tribal communities’ distrust of researchers and data collection processes resulting in a lack of reporting. The large loss of youth to suicide is devastating to AI/AN communities and obstructs Indigenous values and visions of youth as sacred and future leaders [3].

Compounded by a federal system of severely underfunded mental health care [4], suicide risks among AI/ANs include but are not limited to: mental health problems, chronic pain [5–7], historical trauma [8–10], adverse childhood experiences [11]—including domestic violence or history of abuse, alcohol or drug misuse [10, 12, 13], and the suicide attempt or death of family or friends [7, 13]. Protection from suicide for AI/AN communities has been linked to cultural factors, including tribal spirituality, participation in cultural activities, social support from tribal leaders, and holistic connectedness to self/family/community/land [14–18]. Suicide rates also vary greatly based on geographic region and tribal community [19]. While some tribes experience little suicide loss, many impacted AI/AN communities are mobilizing to end this disparity through community-driven innovations and evaluations.

The White Mountain Apache Tribe (WMAT) and Johns Hopkins Center for American Indian Health (JH CAIH) have been research partners addressing suicide prevention since 1994. Self-determination underscores WMAT suicide prevention efforts: In 2002, WMAT passed a tribal resolution mandating the reporting of all suicidal behaviors for all departments within the tribe’s jurisdiction. This resolution includes mandated reporting of suicide ideation, attempts, and deaths, as well as non-suicidal self-injury (added in 2008), and binge substance use (added in 2010) to the Celebrating Life suicide prevention program. This program is led by a group of White Mountain Apache community mental health specialists (CMHSs) using a common registry system (see Cwik et al., 2014 [20]). This community-wide suicide surveillance and case management follow-up system has become a nationally-recognized model with endorsements from the Academy of Child and Adolescent Psychiatry, Indian Health Service, and The Substance Abuse and Mental Health Services Administration [20]. Two noteworthy features of the WMAT suicide prevention system are: a) a focus on community-driven solutions that are adjunct to conventional mental health treatment; and b) the employment of community-based paraprofessionals to fill gaps in services, and improve the cultural congruence and continuity of care [21, 22].

Between 2001 and 2006, data collected through the tribal surveillance system documented WMAT suicide rates at 40.0 per 100,000, nearly 11 times the U.S. All Races rate [23]. The highest suicide rates were among 15 to 24 year olds at 128.5 per 100,000, which was 13 times the U.S. All Races rate and 7 times the All AI/AN rate. Since 2008, the WMAT-JH CAIH research team has completed a series of risk and protective factor studies utilizing qualitative and quantitative approaches that uncovered the critical role of substance use, specifically binge substance use, as a co-occurring risk factor for suicide attempts and deaths. In addition, impulsivity and challenges with family support were dominant risk factors for suicide attempts and related substance use [24]. Research also illustrated positive cultural identity and connectedness to family and community as strong potential protective factors [24, 25].

Based on these findings, the WMAT-JH CAIH research partners have followed a tribal participatory research process to adapt, develop and pilot-test several evidence-based and culturally-informed interventions that form a comprehensive public health approach to suicide prevention [26]. In addition to surveillance and case management, key components of this public health approach include: 1) a culturally adapted brief intervention called “New Hope” focused on safety-planning and suicide risk reduction delivered to youth and a family member by a WMAT CMHS following a suicide attempt [21], and, 2) a culturally grounded, upstream suicide prevention program called “The Elders’ Resilience Curriculum,” (implemented in 2012) in which Elders teach cultural knowledge and values observed as protective against suicide. Between 2007 and 2012 some of these interventions (i.e., New Hope) were implemented in concert with surveillance and case management, broad-based community education, awareness, and outreach about suicide prevention (e.g., door-to-door campaigns and workshops focused on mental health awareness, parenting, and other topics), and a range of other interventions (see Cwik et al., 2016 for a complete description [27]). During that time period, there was a 38.3% reduction in suicide deaths among WMAT community members of all ages and a 23% reduction among WMAT youth ages 15–24, compared to the previous 6 years in the same population and during a period when suicide was increasing or staying the same among other AI/AN same-age and U.S. All Races populations. During this same time period, the annual number of suicide attempts also decreased from 75 (in 2007) to 35 (in 2012) for the entire WMAT population [21].

These promising trends prompted the WMAT-JH CAIH team to pursue a rigorous evaluation of the “New Hope” and “Elders’ Resilience” interventions in tandem with the existing surveillance and case management system. A Sequential Multiple Assignment Randomized Trial (SMART) design was collaboratively selected as the most appropriate study design by WMAT-JH CAIH partners. A SMART design provides data that can be utilized to create adaptive interventions targeting mental health prevention and treatment [28]. The SMART design will answer the primary study aim: how to combine three promising interventions (New Hope, Elders’ Resilience, and Optimized Case Management) for youth after a suicide attempt, suicide ideation, or binge substance use with recent (past 3 months) suicide ideation.

Our primary research questions include: 1) What is the added impact of New Hope on reducing suicide ideation? 2) What is the added impact of Elders’ Resilience on promoting resilience factors? 3) What is the added impact of New Hope and Elders’ Resilience on youth suicide risk and resilience? 4) How does Optimized Case Management compare to the brief intervention combinations? And, 5) Which intervention or combination of interventions works best to reduce suicide ideation and increase resilience based on age and presenting suicidal-behavior event type (e.g., youth with suicide ideation vs. suicide attempt vs. binge substance use with recent suicide ideation)?

This study is part of a larger collaborative hub, including four additional tribes in the Southwest (5U19MH113136–03) and one in Montana, focused on reducing suicide among youth in their communities [29]. WMAT serves as the research intensive partner in the collaborative hub, and if the brief interventions are proven effective by this SMART, there is great potential for adaptation and replication with the five tribal hub partners, and ultimately, other tribes across Indian Country.

## Methods/design

### Overview of study design

**Fig. 1:**
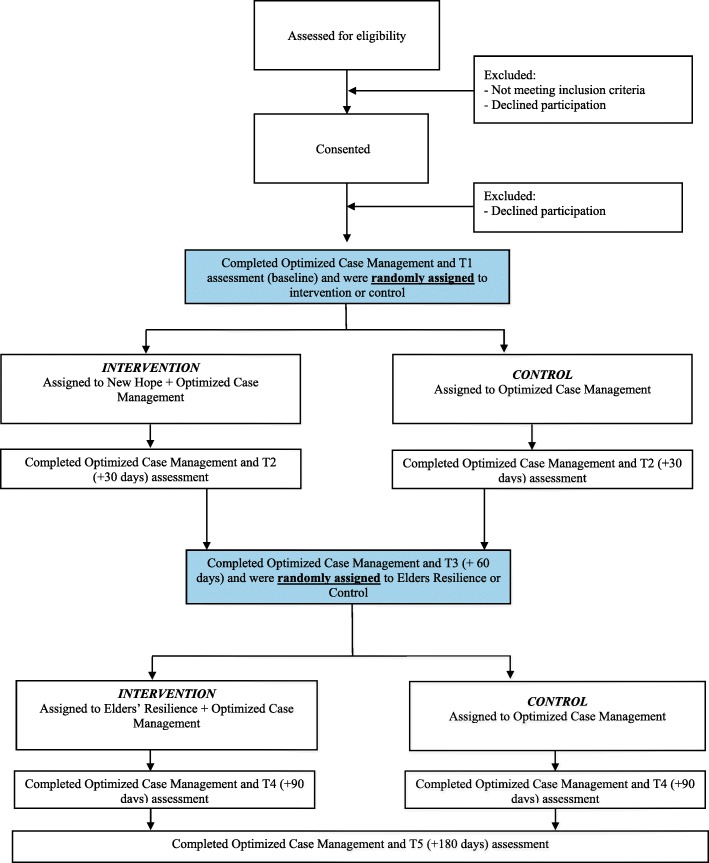
Trial Design

The study utilizes a SMART design whereby eligible youth are randomized at two timepoints to one of two conditions. After an initial baseline assessment, participants are randomized to either New Hope or “Optimized Case Management” (Note: we describe below what we mean by Optimized Case Management intended to meet safety and ethical standards of the research). The second randomization timepoint is at 60 days post baseline assessment timepoint, when participants are then re-randomized to either participate in Elders’ Resilience or Optimized Case Management. All randomization is done using blocking based on age group and type of suicidal behavior to help ensure equal distribution of ages and types of behaviors in all groups (See Fig. 1).

Sequential Multiple Assignment Randomized Trials (SMART) are primarily aimed at learning about optimal sequencing of interventions in order to inform optimized Adaptive Treatment Strategies (ATS) to meet an individual’s needs [30]. Our trial employs a SMART design with the goal of determining how combinations of promising interventions (Optimized Case Management, New Hope, Elders’ Resilience) can prevent suicide among WMAT youth and for which subgroups of youth these combinations are most useful (See Fig. 1). Our study is designed to result in four statistically comparable groups: 1) New Hope plus Optimized Case Management; 2) Elders’ Resilience plus Optimized Case Management; 3) New Hope plus Elders’ Resilience plus Optimized Case Management; and 4) Optimized Case Management alone. We will determine which group improves the most on suicide ideation and resilience outcomes, ultimately informing an adaptive intervention approach that combines these brief interventions based on empirical findings to have the biggest impact on suicide outcomes. Our hypothesis is that suicide risk will reduce and resilience will increase for all groups, but the largest risk reductions will be for youth who receive New Hope, while resilience will increase the most among those youth who receive Elders’ Resilience. We also hypothesize that the largest changes in risk and resilience outcomes will be for youth who receive both New Hope and Elders’ Resilience compared to youth who receive Optimized Case Management alone.

**Fig. 2:**
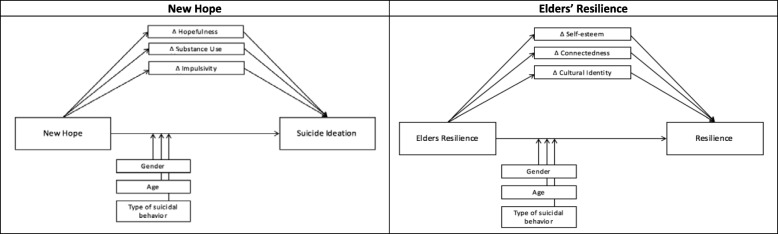
Hypothesized Exploratory Mediation and Moderation Models

We will also explore possible mediators and moderators of intervention effects. Selection of mediators and moderators was guided by WMAT-JH CAIH previous research [24, 31, 32]. Moderators including age, gender, and event type (i.e., suicide ideation, binge substance use with recent suicide ideation, and suicide attempt), are exploratory to determine which types and sequence of interventions are best suited for which youth. We also hypothesize that hopefulness, substance use, and impulsivity will mediate the relationship between New Hope and suicide ideation due to the focus on increasing coping and self-regulation skills (See Fig. 2) [24, 31, 32]. Given the strengths- and culturally-based focus of Elders’ Resilience, we hypothesize that self-esteem, connectedness, and cultural identity will mediate the relationship between this intervention and resilience (See Fig. 2).

### Study site

This study will be conducted by the WMAT and JH CAIH research team. The WMAT resides on the Fort Apache Indian Reservation in Eastern Arizona. There are 25 distinct communities within the reservation. The WMAT is governed by a Tribal Council that includes a Tribal Chair, Vice Chair, and 9 Council Members. The WMAT includes approximately 17,500 enrolled tribal members with more than one-third youth ages 10 to 24. This study was approved by the Tribal Council, Tribal Health Advisory Board, and Johns Hopkins University research review boards (Current protocol version: #8, IRB#00008138). Any modifications to the study protocol are submitted as an amendment to relevant IRBs. This manuscript was approved by the Tribal Council and Tribal Health Advisory Board.

### Participants

Participant inclusion criteria includes: 1) AI youth 10 to 24 years old; 2) residing on or near the Fort Apache Indian Reservation; 3) written consent for those over age 18, plus child assent and parent/guardian consent for youth under 18 years old; and 4) suicide ideation in the past 30 days, suicide attempt in the past 30 days, or binge substance use in the past 30 days with suicide ideation in past 3 months (*Note: as described in the background section, binge substance use and suicide-related behaviors often co-occur in this community and are a significant risk for future suicidal behavior including death*), as identified and verified by the WMAT suicide surveillance system [20]. As specified by the tribe’s surveillance system, suicide ideation is defined as thoughts to take one’s own life with or without preparatory action; binge substance use is defined as consuming substances with the intention of modifying consciousness and resulting in being found unresponsive or requiring Emergency Department treatment; and suicide attempt is defined as intentional self-injury with the intent to die (includes interrupted or aborted suicide attempts by self or others). Study exclusion criteria includes: 1) unstable and severe medical, psychiatric, or substance use problem that requires inpatient treatment; 2) acute suicidal or homicidal ideation requiring immediate intervention; 3) recent severe distressing life events (e.g., physical or sexual abuse or violent victimization) that requires specific and immediate intervention or out of home placement; 4) severe visual impairment; 5) not speaking English; and 6) any other condition that renders an individual without full capacity to provide informed consent.

### Recruitment and consent

Participants are recruited through the WMAT suicide surveillance system (locally known as “Celebrating Life”). When community members are identified through the surveillance system, a WMAT Celebrating Life team member conducts an in-person follow-up visit at the individual’s home or another private setting to verify the event, collects information about the individual, and provides referrals to the local mental health center as well as religious or spiritual-based services or traditional healing if requested [20]. During the follow-up visit, the Celebrating Life team member assesses study eligibility using an approved script. If the individual meets inclusion criteria and is interested in participating, a WMAT research staff member obtains informed consent/assent. Some of the research staff are in the role of Independent Evaluators (IEs) and assist with administering self-report assessments via a tablet with audio assistance while providing Optimized Case Management to all participants; IEs do not learn or assist with administering the brief interventions. Other research staff are in the role of Interventionists, and deliver New Hope and support Elders delivering Elders’ Resilience. Interventionists do not administer study outcome assessments, but they provide a session summary sheet for each visit. All research staff have been trained in ethical considerations and study procedures. In order to be certified as study ready, they had to pass a knowledge test on study procedures, scoring > 80%. Interventionists had to pass an additional written test, also scoring > 80%, specific to delivering the New Hope intervention. Elders who already deliver a multi-session version of the Elders’ Resilience intervention in local classrooms were trained in how to deliver the brief, individually-based Elders’ Resilience intervention specific to this study and were trained in ethical considerations.

### Randomization and blinding

When eligible participants are identified by the surveillance system and consent/assent is obtained, an IE administers the baseline assessment. Baseline and all follow-up assessments are self-report assessments via a tablet using REDCap with audio assistance and administered by the IE. At the completion of the baseline assessment, participants are randomized 1:1—stratified by age group (10–14, 15–19, 20–24) and event type (suicide ideation, binge substance use plus recent suicide ideation, suicide attempt)—to either the control condition, Optimized Case Management alone, or New Hope plus Optimized Case Management. Re-randomization of all participants occurs again at Visit 3 (60 days post baseline assessment and after New Hope has been administered to the intervention group) (see Fig. 1). Initial randomization (i.e., New Hope/Optimized Case Management) is done via a random number generator in REDCap and results are not displayed to keep the assessors blinded to study status. A data manager reviews the results each day to inform the Interventionists who is randomized to the intervention groups so that a visit can be scheduled. Randomization assignments for the second sequence of the intervention (Elders’ Resilience/Optimized Case Management) is done using a random number table in Microsoft Excel. The results of the second randomization are entered into REDCap by the data manager for each participant. The data manager then informs Interventionists who is randomized to the intervention group so that an intervention visit can be scheduled.

### Sample size

The power calculation for this study was completed for the primary outcome of suicide ideation. Based on previous work using the Suicide Ideation Questionnaire (SIQ), a 25% reduction among individuals with a score of 27 or greater for individuals over age 16, and 16 or greater for youth 15 and younger (clinically significant cut-off score in local population [33]) would represent clinically significant change in suicide ideation [27, 34]. Pilot data of the New Hope intervention with WMAT youth showed an average baseline score of 45 could be expected on the SIQ. This clinically significant reduction translates into a large Cohen’s D effect size (> 0.8). However, we wanted power to observe the more moderate effects expected from comparing the sequencing of the four combinations of interventions. Using an adjusted alpha to account for multiple comparisons of 0.013, five study assessment points with a correlation of 0.2 among repeated measures, and four groups, resulted in a sample size of 58 participants per group in each of the four groups at end point, to provide 80% power to detect a small to moderate effect (Cohen’s F = 0.2). Adjusting these estimates for 30% attrition resulted in the need to recruit *n* = 76 prospective participants for each of the four groups for a total sample size of *N* = 304 to be enrolled at baseline.

### Intervention

#### Optimized Case Management

IEs are blinded to study assignment, administer all assessment batteries (self-report measures via a tablet using REDCap supplemented with audio assistance), and complete Optimized Case Management. Optimized Case Management visits occur in the participants’ home or another private setting on a monthly basis during the study period for all participants. At the beginning of each Optimized Case Management visit, the study assessment is administered, which includes the Suicide Ideation Questionnaire (SIQ) for participants ages 15 to 24 and the Suicide Ideation Questionnaire – Junior (SIQ-JR) for participants ages 10 to 14 to assess imminent risk [34]. If the youth is deemed to be at imminent risk for suicide based on the SIQ/SIQ-JR score from the assessment battery, the IE follows procedures according to an established suicide risk protocol we detail below. Consistent with standard case management, the IE builds rapport with the participant, connects the youth to behavioral health and/or other available community services (e.g., traditional healer, religious-based services, social services) and, participants may request additional case management visits as needed between assessment visits. Any additional case management visits will be documented and considered in the final analysis.

#### New Hope

New Hope is based on a brief empirically-validated Emergency Department intervention that underwent deep structure adapation by the WMAT-JH CAIH study team in 2008 [35–38] (see Cwik et al., 2016 [27]). Consistent with the previous pilot period, New Hope is delivered in this study by a trained Apache CMHS in one visit over 2 to 4 h in a private setting. Youth are invited to include a family member to participate in the intervention to provide support and reinforce the skills learned. New Hope emphasizes the seriousness of suicide ideation, attempt and/or binge substance use with recent ideation, teaches coping skills (e.g., emotion regulation, cognitive restructuring, increasing social support) and suicide safety planning, and aims to reduce barriers to treatment motivation, initiation, and adherence. The intervention includes a 20-min video produced by WMAT-JH CAIH featuring AI actors portraying scenes specific to the characteristics of suicide attempts, ideation and related binge substance use among youth ages 10–24 in this community, with WMAT Elders speaking in Apache (with subtitles) about how life is sacred, how suicide is not the Apache way, how self-harm impacts the entire community, their concern for the youth, and the importance of each youth’s life. Pilot data with WMAT youth who had recent suicide attempts and received New Hope showed decreases in self-reported depressive symptoms, suicide ideation, negative cognitive thinking and Emergency Department visits for behavioral and mental health, and increases in youth connecting with mental health care services [27].

#### Elders’ Resilience

For many years, the study team’s local Community Advisory Board, Elders’ Council, Tribal Council, and Tribal Health Board have emphasized the importance of Apache traditions, cultural beliefs and values to prevent youth suicide. Beginning in 2011, WMAT and JH CAIH worked together to develop a monthly manualized school-based curriculum delivered by WMAT Elders to youth as an upstream, culturally grounded suicide prevention intervention [39]. In the Apache language, the curriculum is called Nowhi nalze’ dayúwéh bee goldoh dolee, with an approximate English translation to “Let our Apache heritage and culture live on forever and teach the young ones.” Each lesson is comprised of Elders introducing youth to Apache language, cultural knowledge, and stories, with a strong focus on respect and the sacredness of life. The curriculum is organized by once-a-month 30-to-45 min lessons, with each month featuring specific themes (e.g., respect, self-worth, spirituality, relationships/clan system) and relevant seasonal teachings. Pilot data analysis for the school-based curriculum is underway. The WMAT-JH CAIH research team worked closely with Apache Elders to adapt the monthly school-based curriculum into a single 2 to 4 h session. This shortened version was developed due to Elders and Tribal Council expressing a desire to meet on a one-on-one basis with at-risk youth in their homes to teach these cultural lessons with youth at high risk for suicide and their families. This brief version of Elders’ Resilience is being delivered in the current study by an Elder with support from one of the WMAT Interventionists. The curriculum covers strength through prayer and tradition, respect, your journey, connection with our community, our clans, our traditions and a promise for the future, and includes working collaboratively on a piece of traditional jewelry that the youth keeps.

### Outcomes

#### Primary outcomes

The primary outcomes for this study are 1) **suicide ideation** as measured by the SIQ/SIQ-JR, and 2) **resilience** as measured by a modified version of the Resiliency Scales for Children and Adolescents (RSEA) (see Table 1 for full list of measures and assessment timepoints) [33, 34, 40]. The SIQ and RSEA are completed at baseline, immediately following completion of New Hope (30 days post baseline), at 60 days post baseline, immediately following completion of Elders’ Resilience (90 days post baseline), and again at 120 and 180 days post baseline for all groups.

**Table 1:** Assessment Table and Timepoints

	T1 (Baseline)	T2 (+ 30 days)	T3 (+ 60 days)	T4 (+ 90 days)	T5 (+ 180 days)
Demographics	X		X		X
Suicide Ideation Questionnaire (SIQ/SIQ-JR) [33, 34]	X	X	X	X	X
Resilience Scales (MAS, REL, REA) [40]	X	X	X	X	X
Centers for Epidemiologic Studies Depression Scale Revised (CESDR-10) [41]	X	X	X	X	X
The Children’s Hope Scale [42]	X	X	X	X	X
The WHO Alcohol, Smoking and Substance Involvement Screening Test (ASSIST) [43]	X	X	X	X	X
UPPS Impulsive Behavior Scale [44]	X	X	X	X	X
Multicultural Mastery Scale [45]	X	X	X	X	X
Voices of Indian Teens Cultural Issues and Interest [46–48]	X	X	X	X	X
Rosenberg Self-Esteem Scale [49]	X	X	X	X	X
Index of Local Indicators of Well-Being	X	X	X	X	X
PROMIS Pediatric Anxiety Short Form [50]	X	X	X	X	X

#### Secondary outcomes

Secondary outcomes for this study (See Table 1) include depressive and anxiety symptoms, impulsivity, self-efficacy and communal mastery, importance of following AI values and cultural practices, self-esteem, hope, substance use, and a subset of local WMAT generated items relating to changes observed among youth who have previously received New Hope and/or Elders’ Resilience interventions (See next section for description of how these items were generated).

#### Selection and adaptation of measures

Prior to starting the study, we worked with our local WMAT collaborators to review all study measures and the face validity of each item. Collaborators were WMAT members of the WMAT-JH CAIH research team who had worked with individuals experiencing suicide ideation/behavior(s) for more than 2 years and were involved in implementing a variety of suicide prevention and youth enrichment programs. They identified several items on the resilience scales and the SIQ that did not seem culturally relevant. For example, the SIQ asks about whether the individual has a will. However, drafting wills is not a common practice in this community. Other items were reworded slightly to be more literal. Finally, response options were changed to reflect local ways of expressing levels of severity while maintaining fidelity to the structure of the scales. For example, on the resilience scale, the response options of 0 “Never” to 3 “Almost Always” were changed to 0 “Not at all” to a 3 “A lot.”

We also used nominal group and free-listing techniques to identify other potential intervention effects not captured by standard assessment instruments. As both the New Hope and the longer Elders’ Resilience interventions had been piloted previously in this community and delivered by WMAT CMHSs participating in assessment review, they had personally observed changes youth experienced as a result of these interventions that were not perceived to be measured by the assessment battery. To capture these, we used a free listing activity based on prior work by Bolton and colleagues to better understand intervention impacts [51]. WMAT CMHSs were asked two questions: “What are all the changes that a youth experiences because they participated in New Hope?” and “What are all the changes that a youth experiences because they participated in Elders’ Resilience?” CMHSs generated as many answers as they could on their own sheets of paper, and then we compiled a comprehensive list of these outcomes. Next, we used this list to re-examine all assessment items. Where an item overlapped with a concept in the existing assessment battery, we noted it. We then created a list of local indicators of well-being not captured in existing assessments and created an index of 11 locally generated questions to monitor change on these tribally-specific derived outcomes (See Table 1).

### Monitoring of intervention and data quality

Data quality review occurs on a weekly basis throughout data collection to identify and reduce to the lowest level possible data missingness, inconsistencies and data entry mistakes. Our data system, REDCap, automatically generates a weekly report that identifies all concerns by concern type and participant ID. This information is then given to IEs to correct, or follow-up with the respondent. In addition to weekly data quality scans, the IEs answer a brief “session summary” form after each evaluation that addresses other data quality concerns. For example, IEs report on the number of interruptions that took place during the interview, the number of individuals present during data collection, and if they knew the participant prior to data collection. This information is collected with the aim of continually monitoring and training IEs and controlling for any protocol departure variables in the final analyses stage.

### Safety of participants

The WMAT-JH CAIH team has an established suicide risk safety protocol that is utilized in this study. During all assessment and intervention visits (Optimized Case Management, New Hope, or Elders’ Resilience), every participant receives the Suicide Ideation Questionnaire (SIQ/SIQ-JR) [34] to determine current suicide risk and ensure participant safety. If the SIQ score is above 37 and SIQ-JR score is above 16, WMAT study staff administer the SIQ-Past Few Days (SIQ-PFD) that includes 6 critical items to further assess frequency and severity of suicide ideation in the last few days. Based on the participants’ SIQ/SIQ-JR score, they are categorized into one of three categories: 1) does not appear at risk for suicide (SIQ below 37 or SIQ-JR below 16); 2) at some risk for suicide (SIQ above 37 or SIQ-JR above 16 and 0 items scored 4 (several times in the past few days), 5 (once a day), or 6 (several times a day) on SIQ-PFD); or 3) at medium to very high risk for suicide (SIQ above 37 or SIQ-JR above 16 and at least 1 item scored 4, 5, 6 on SIQ-PFD). These cut-off scores are based upon previous research with the WMAT community to accurately detect suicide ideation and suicide attempts [33].

If the participant falls into the “does not appear at risk for suicide” category based upon SIQ/SIQ-JR score and their own observations when interacting with participants, study staff do not take immediate action. If the participant is deemed to be “at some risk for suicide” study staff call the WMAT Behavioral Health Program Manager on site to review the case and develop a plan, including assessment and referral to local tribal behavioral health services. The Behavioral Health Program Manager notifies either one of the Co-PIs or a Co-I (two are Licensed Psychologists) to review and confirm the plan. If the participant is “at medium to very high risk for suicide”, study staff call the WMAT Behavioral Health Program Manager to review the case including SIQ scores, family support, determine if the participant has an upcoming behavioral health appointment, and develop a plan which may include an urgent appointment with local tribal behavioral health services or Emergency Department visit if the participant is at imminent risk for suicide. The Behavioral Health Program Manager notifies one of the Co-PIs or a Co-I (two are Licensed Psychologists) to review and confirm the plan; parent/guardian(s) are also notified if immediate triage of participants is necessary to ensure their safety.

Serious adverse events (e.g., participant death) for youth participants are reported in real-time to participating IRBs and the trial’s Data Safety and Monitoring Board (DSMB). The DSMB includes three members: one suicide prevention expert, one biostatistican, and one expert in AI/AN health. The three-member DSMB meets with study investigators biannually and reviews trial progress and any adverse events. The trial data team unblinds adverse events for review by the DSMB; however, the PIs do not have access to this information. If the DSMB were to determine any interventions were causing harm to participants, the intervention and/or study would be revised or discontinued.

### Statistical analysis

#### Primary aim

Our data analysis plan is designed to determine which of the four sequences of interventions: New Hope plus Optimized Case Management, Optimized Case Management plus Elders’ Resilience, New Hope plus Elders’ Resilience plus Optimized Case Management, and Optimized Case Management alone have the greater effects on suicide ideation and resilience 6 months post-index events (baseline). Given the longitudinal nature of the data, we will use mixed-effects regression models (MRMs) to estimate average treatment effects over 6 months while accounting for shared variance of repeated measures within participant [52]. MRMs are highly flexible, addressing: variability in number and spacing of measurements, a variety of outcome distributions, linear and non-linear patterns of change (e.g., quadratic), and are robust to missing data.

The use of MRMs will also allow for testing piecewise or discontinuous change to obtain highly targeted tests of intervention effects [53]. Specifically, this technique will involve adding a time-varying dichotomous indicator for participants receiving New Hope in the initial randomization, and individuals receiving Optimized Case Management or Elders’ Resilience in the second randomization. With these indicators simultaneously modeled, the reference category will represent individuals receiving Optimized Case Management in the first randomization and will allow us to test for change in means across all four groups. For example, this coding strategy will allow testing for a change in means for individuals receiving New Hope followed by Optimized Case Management and individuals receiving New Hope followed by Elders’ Resilience.

#### Secondary aim

Due to anticipated sample sizes, proposed mediation and moderation models are hypothesis generating only. Proposed moderators (i.e., suicide ideation, binge substance use with recent suicide ideation, and suicide attempt) will be evaluated by including them at the appropriate level of measurement in the models detailed above, and interaction terms will be specified between moderator and intervention terms of interest.

For the proposed mediators (hopefulness, substance use, and impulsivity for New Hope; and self-esteem, connectedness, and cultural identity for Elders’ Resilience), a key strength is that the mediator and outcome processes are measured longitudinally; however, this approach requires a specialized model for testing mediation [54]. A parallel process latent growth curve mediation model will be used to simultaneously model change over time in the mediator and change over time in the outcome, where for the mediation test, the mediator slope will be specified as a predictor of the outcome slope. The statistical test for the mediated effect will be based on the product of the coefficients for the extent to which the intervention changes the mediator and the extent to which the mediator affects the outcome (i.e., αβ). We will calculate asymmetric bootstrapped *SE*s and confidence intervals for the mediation effect to determine precision. Use of a parallel process latent growth curve model offers greater statistical power relative to traditional causal steps approaches [55].

## Discussion

This study is groundbreaking in its use of an innovative evaluation design to understand how brief interventions used alone or in sequence can prevent suicide ideation and behaviors among an AI youth population. In addition, the prevention interventions have been specifically designed to reach youth outside of the mental health system—which presents formidable barriers to care, and are delivered by local AI CMHSs, increasing capacity for mental health care, sustainability and, ultimately, potential scaling to other communities with few mental health care resources [22, 56]. The interventions have been adapted or co-created through a strong tribal-academic partnership to target locally relevant modifiable risk and protective factors for suicide and related behaviors. We are also focused on testing **brief** interventions, to build on the experience of clinicans in this field that some youth may only need short-term assistance to overcome a period of intense emotional discomfort and can learn skills and integrate concepts and new perspectives to protect them during future periods of distress. Our study will contribute to the growing literature examining brief suicide prevention interventions [57]. However, our focus on testing brief suicide prevention interventions delivered to AI/AN youth by CMHSs or local Elders in community settings is novel. To move the field forward, the use of a SMART evaluation design is powered to detect which *combination of brief interventions* works to reduce suicide ideation and promote resilience, while our moderation analysis will allow us to explore what type of individuals—considering age and type of index event—benefit most from which intervention(s). Ultimately, this study seeks to inform the creation of a scalable, adaptive model to reduce suicide risk and promote resilience among AI youth.

### Limitations

Studies of youth suicide are inherently challenging due to the critical need for ensuring the safety of all participants while developing evidence for what interventions are more or less effective [58]. The selection of the SMART design was aimed at increasing opportunities for participants to be exposed to one or two promising brief interventions while maintaining and optimizing case management as the standard of care for all participants, since case management has proven to be an effective approach to help reduce suicide in this community [21]. Optimized Case Management may dilute our ability to detect between group differences and we cannot detect independent effects of Optimized Case Management alone, without a treatment-as-usual control group. However, Optimized Case Management is necessary to ensure safety of participants by optimizing the current highest standard of care and prevention in the community. The WMAT-JH CAIH research team also has a well-established suicide risk protocol to address safety concerns of participants being used in this study. Finally, because this study was designed to produce knowledge about suicide prevention building on a long line of research with one Southwest AI community, results may not generalize to other tribal communities. However, other components of the grant funding this work (5U19MH113136–03) and additional grants (NIMH K01 MH116335) are focused on determining how the interventions, if proven effective, can be adapted and scaled to other tribal communities who have key stakeholders participating on a cross-site steering committee that receives quarterly briefings about this research and additional technical assistance to prevent suicide in their own communities [29].

### Strengths

This study answers a call for empirical research to evaluate protective factor and culturally-based interventions to address AI/AN youth suicide [59, 60]. WMAT partners have dedicated years of effort, resources, and time to develop community-driven interventions to reduce suicide in their own community. Community mental health specialists (i.e., paraprofessionals) are used at all levels of this study; from the Interventionists who are delivering the intervention, to the co-investigators who are leading overall grant efforts. AI paraprofessionals play an important role in carrying out community-led interventions that empower the community, uphold tribal sovereignty, and build capacity to sustain efforts long-term [61]. If study results are achieved, there is promise for lasting benefits for the community, other tribal nations, and significant advances for the suicide prevention field.

## Data Availability

Not applicable.

## Abbreviations

AI/AN: American Indian/Alaska Native
ATS: Adaptive Treatment Strategies
CMHS: Community Mental Health Specialist
IE: Independent Evaluator
JH CAIH: Johns Hopkins Center for American Indian Health
MRM: Mixed-effects regression models
RSEA: Resiliency Scales for Children and Adolescents
SIQ: Suicide Ideation Questionnaire
SIQ-JR: Suicide Ideation Questionnaire – Junior
SIQ-PFD: Suicide Ideation Questionnaire – Past Few Days
SMART: Sequential Multiple Assignment Randomized Trial
T1: Timepoint 1
T2: Timepoint 2
T3: Timepoint 3
T4: Timepoint 4
T5: Timepoint 5
U.S: United States
WMAT: White Mountain Apache Tribe
